# A Systematic Review of Phase II Targeted Therapy Clinical Trials in Anaplastic Thyroid Cancer

**DOI:** 10.3390/cancers11070943

**Published:** 2019-07-04

**Authors:** Josip Ljubas, Therese Ovesen, Maria Rusan

**Affiliations:** 1 General Practice, The Doctors of Viborg Stadion Allé 1, 8800 Viborg, Denmark; 2 Department of Clinical Medicine, Aarhus University, 8200 Aarhus, Denmark; 3 Department of Otorhinolaryngology, Region Hospital Holstebro, 7500 Holstebro, Denmark; 4 Department of Clinical Pharmacology, Aarhus University Hospital, 8000 Aarhus, Denmark

**Keywords:** anaplastic thyroid cancer, thyroid neoplasms, targeted therapy, clinical trials, precision medicine

## Abstract

Anaplastic thyroid carcinoma (ATC) is a rare, but devastating disease. Despite multimodal approaches combining surgery, chemotherapy and radiation therapy, ATC is associated with a dire prognosis, with a median overall survival of only three to ten months. Novel treatments are thus urgently needed. Recent efforts towards the characterization of the molecular landscape of ATC have led to the identification of pro-oncogenic targetable alterations, lending promise for novel targeted therapeutic approaches. This systematic review summarizes the results of phase II clinical trials of targeted therapy in ATC, providing an overview of efficacy and safety profiles. The majority of trials to date have consisted of small single-arm studies and have presented modest results. However, only a minority of trials have selected or stratified patients by molecular alterations. In the setting of BRAF V600E mutated ATC, dabrafenib/trametinib combination therapy and vemurafenib monotherapy have both demonstrated efficacy. Everolimus has furthermore shown promising results in patients with PI3K/mTOR/AKT pathway alterations. These studies underscore the importance of molecular profiling of tumors for appropriate patient selection and determination of genomic correlates of response. Clinical trials are underway testing additional targeted therapies as monotherapy, or as a part of multimodal treatment, and in combination with immunotherapy.

## 1. Introduction

Anaplastic thyroid carcinoma (ATC) is a rare and aggressive type of thyroid cancer, accounting for only 2% of all thyroid carcinoma (TC) cases [1], yet for more than half of TC-related deaths. It is associated with a median overall survival (OS) of 3–10 months, a median 1-year survival of 20%, and a median 10-year OS of less than 2% despite aggressive multimodal management [2,3,4,5,6]. 

### 1.1. Clinical Presentation and Histopathology

ATC commonly presents in the 6th and 7th decade of life. Only 10% of patients present with local disease, 40% present with loco-regional disease and 50% present with distant metastasis [7]. Due to the aggressive clinical course, The American Joint Committee on Cancer has categorized all ATC tumors as stage 4 tumors (T4); T4a representing local disease, T4b representing loco-regional disease and T4c representing distant metastasis [8]. ATC arises from follicular thyroid cells, and is the most dedifferentiated subtype of TC, not retaining any of the biological features of follicular cells. It has been suggested that thyroid cancer stem cells, may also play a role in ATC tumor initiation and growth, and may confer an aggressive phenotype and resistance to chemo- and radiation therapy [9,10]. ATC is heterogeneous with varying mixtures of spindle cells, squamous cells and pleomorphic giant cells [11] and is characterized by extensive infiltration of macrophages, which have also been suggested to promote the aggressiveness of the disease [12]. 

### 1.2. Pathogenesis

The tumorigenesis of ATC is not well understood. Most studies support a stepwise dedifferentiation from well-differentiated (WDTC) to more poorly differentiated thyroid carcinoma (PDTC), and finally to ATC, with the progressive accumulation of somatic pro-carcinogenic mutations. This is supported by the fact that 18–37% of ATC cases arise from longstanding goiters [13,14,15,16,17,18], WDTC lesions and ATC co-occur in 30–89% of cases [13,19,20,21,22], and ATC sometimes develops following treatment failure of WDTC and PDTC [23]. Genomic analyses have further demonstrated shared mutations between co-existing ATC and WDTC or PDTC lesions, suggesting a common originating cell [24,25]. Nevertheless, some evidence suggests that at least a proportion of ATC cases can arise independently with no identifiable pre-existing or co-existing more differentiated lesion [25,26]. 

### 1.3. Molecular Landscape of ATC

The molecular landscape of ATC has been described in several recent comprehensive reviews [11,27], primarily summarizing the findings of three recent next generation sequencing (NGS) studies [24,28,29]. Several additional NGS studies have since emerged [25,30,31,32,33,34,35]. These findings are briefly summarized below, and in Table 1. Discrepancies between studies may largely reflect differences in methodologies.

In comparison to more differentiated TC, ATC is associated with a higher mutational load [24,25]. The most frequently identified alterations include telomerase reverse transcriptase (*TERT*) promoter mutations and *p53* mutations, and these alterations are far more common in ATC than in PDTC, suggesting a role for these in further dedifferentiation [24,25,30,31]. Activating *TERT* mutations, specifically in C228T and C250T, have been suggested to create de novo binding elements for E26 transformation-specific (ETS)-family transcription factors responsive to MAPK signaling [24]. Additionally, mutations in both the RAS-RAF-MAPK pathway and PI3K-AKT-mTOR pathway have been found to be enriched in ATC. *BRAF* mutations, in particular BRAF V600E, as well as RAS mutations are common, with BRAF V600E mutations being more frequent in ATC compared to PDTC. Interestingly, *BRAF* and RAS mutations have been noted to be mutually exclusive [24,25,28,29]. Furthermore, mutations in members of the SWI/SNF chromatin remodeling complex, such as *ARID1A*, *SMARCB1*, and *ATRX*, have been identified in up to 36% of cases, and appear to be mutually exclusive, suggesting that mutations in any one gene are sufficient to impair function [24]. Additionally, mutations of histone methyltransferases and mismatch repair genes have frequently been noted, as well as alterations in cell cycle genes [24,25,28,29,30,32,34,35]. The latter primarily include loss-of-function mutations in *CDKN2B* and *CDKN2A* and amplification of *CCNE1* [25,30,31,32,35]. Cell cycle gene alterations have been identified in 29 % of ATCs vs. 13 % of WDTCs, suggesting a possible role in dedifferentiation [25]. Mutations have been further identified in the Eukaryotic Translation Initiation Factor 1A, X-linked (*EIF1AX*), a component of the translational preinitiation complex, in up to 14% of cases. *EIF1AX* mutations are strongly associated with RAS mutations [24]. Furthermore, amplification of tumor immune-evasion genes such as *PD-L1*, *PD-L2* and *JAK2* has been noted in 5% of tumors [25,32]. Lastly, *ALK* fusions and mutations have been reported in several studies with a frequency of 0–2% [24,25,28,29,31], and up to 11% [36] and 20% [33] in two other studies.

Copy number alterations in ATC are widespread. In particular, 8p and 17p loss and 20q gain have frequently been reported, and loss of 13q and gain of 20q has been associated with decreased survival (log-rank, *p* = 0.02 and 0.06, respectively) [24]. 

Interestingly, Pozdeyev et al. identified four different clusters of ATCs with distinct genetic profiles, three of which resembled dedifferentiation from more well-differentiated TC types and one of which did not [25]. These data support the theory that a proportion of ATC cases represent a dedifferentiation from preexisting TC tumors, while a smaller subgroup may arise independently. 

The literature characterizing gene expression and proteomics in ATC remains scarce. Overexpression of Epidermal Growth Factor Receptor (EGFR) has been noted in 58–87% of ATC tissues, and 10- to 30-fold overexpression of Platelet-Derived Growth Factor Receptor (PDGFR) has been noted compared to normal thyroid tissue [2,37]. Further studies are needed to validate these aspects. Furthermore, thyroid tumors are generally highly vascularized and overexpress Vascular Endothelial Growth Factor (VEGF) [38], and the role of both VEGF and Vascular Endothelial Growth Factor Receptor (VEGFR) in WDTC is well-described, with overexpression of either being linked to poorer prognosis [39,40,41]. VEGF overexpression has also been noted in ATC [42,43]. Some studies have characterized gene expression in a subpopulation of ATC cells, defined as cancer stem cells, finding elevated Notch, Wnt/β-catenin, and Hedgehog signaling, as well as upregulation of NF-κB and JAK/STAT signaling. As this subpopulation of cells has different characteristics than the remainder of the tumor, it has been proposed that it needs to be specifically targeted alongside conventional treatment of the tumor to achieve greater therapeutic efficacy [9,10,44,45]. 

Further investigations are required to delineate the molecular profiles associated with distinct histological subtypes of ATC, as well as to characterize tumor heterogeneity in ATC, and to determine the prognostic significance of distinct alterations. 

### 1.4. Treatment

The current management of ATC consists primarily of surgical resection, combined with adjuvant chemoradiation [46]. Gross resection is, however, limited to a restricted number of patients with T4a (10%) and a small subset of those presenting with T4b disease. Adjuvant chemoradiation with doxorubicin alone, or in combination with taxanes or platins, has been shown to marginally improve survival [5]. Aggressive multimodal therapy with total/near total thyroidectomy (or debulking surgery), cervical lymph-node dissection and chemoradiotherapy, has been shown to improve the outcome of ATC patients with both local and advanced disease, doubling the median OS to 8 months [15]. A retrospective cohort study showed that initial intense multimodal therapy (intensity modulated radiation therapy and chemotherapy, often combined with thyroidectomy) is superior in comparison to palliative intent therapy in patients with advanced ATC, producing a median OS of 21 vs. 4 months, respectively (*p* = 0.0006) [5]. A second retrospective cohort study of ATC patients with all stages of disease showed that treatment with surgery, along with external beam radiation therapy (EBRT) with or without radio-sensitizing or systemic chemotherapy, is superior to dual therapy consisting of EBRT and radio-sensitizing chemotherapy, producing a median OS of 22.1 vs. 6.5 months [47]. A recently published review summarizing different treatment modalities in ATC and their outcomes, concluded that an early multidisciplinary approach using extensive radical surgery, in combination with adjuvant chemo-radiation with docetaxel/paclitaxel or cisplatin provides the best outcomes [48]. Aggressive multimodal treatment comes, however, at the cost of significantly increased toxicity, further underscoring the need for novel therapeutic strategies.

**Figure 1:**
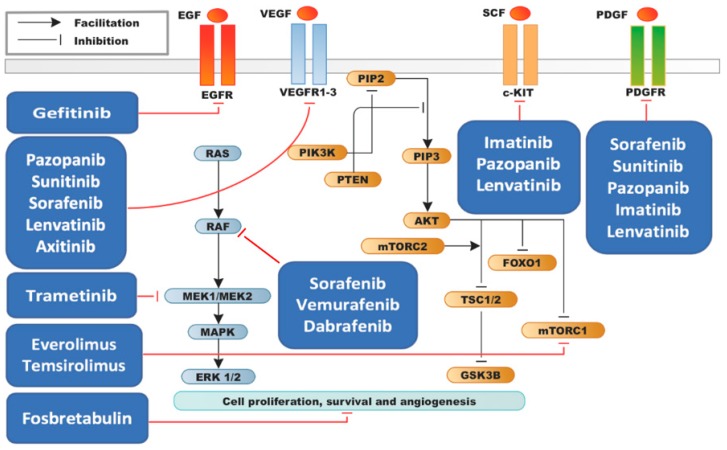
Mechanistic overview of targeted therapy agents tested in ATC.

The recent identification of targetable alterations has led to multiple pre-clinical studies in cellular and mouse models, a number of which showed promising results. These studies paved the way for several clinical trials, the most advanced of which are in phase II, and have led to the approval of the first targeted therapy option for ATC (combination therapy with dabrafenib, a BRAF V600E inhibitor, and trametinib, a MEK1/2 inhibitor). The majority of studies to date have focused on small molecule inhibitors of receptor tyrosine kinases. The agents employed in the studies and their intracellular mechanisms are illustrated in Figure 1.

The aim of this review is to summarize the results of phase II clinical trials of targeted therapy agents in ATC to date, with a focus on efficacy and safety profiles. 

## 2. Results

### 2.1. Efficacy Results of Phase II Clinical Trials

An overview of phase II clinical trials of targeted therapy agents in ATC to date and their efficacy is summarized in Table 2. No phase III clinical trials were identified in our search. Results for the various tested agents are further detailed below.

### 2.2. Multikinase Inhibitors

Sorafenib, an inhibitor of RAF, VEGFR, and PDGFR family kinases, has been tested in a phase II trial in patients with TC, which included a subgroup of four ATC patients. One patient (25%) experienced stable disease (SD) [49]. A second single-arm phase II clinical trial of sorafenib in advanced ATC exhibited partial responses (PR) in two of 20 patients (10%), with response duration of 10 and 27 months respectively [50]. SD was seen in five patients (25%), with a median duration of four months. Median progression-free survival (PFS) was 1.9 months (95% CI: 1.3–3.6 months), and median OS was 3.9 months (95% CI: 2.2–7.1 months). A third single-arm, multicenter study of sorafenib in patients with locally advanced or metastatic TC, which included 10 ATC patients, reported a median PFS of 2.8 months (95% CI: 0.7–5.6) and a median OS of 5 months (95% CI: 0.7–5.7). No PR or complete responses (CR) were achieved, but 40% of ATC patients experienced SD [51]. A fourth phase II trial tested sorafenib in combination with the mTOR inhibitor, temsirolimus, in 36 patients with metastatic, radioactive iodine-refractory TC (2 of which had ATC) [52]. One of the ATC patients experienced PR and was on study for 6.9 months (the other patient had progressive disease (PD)). 

Pazopanib, an inhibitor of VEGFR, PDGFR, Fibroblast Growth Factor Receptor (FGFR) and C-KIT, was tested in 15 patients with advanced ATC with no overall responses seen. Two patients had transient disease regression although insufficient to meet RECIST-criteria. Median OS was 3.7 months and median PFS 2 months [53]. Two patients were still alive, despite PD at 9.9 months and 2.9 years. 

Imatinib, an inhibitor of the Bcr-Abl fusion protein, c-KIT, and PDGFR, has been tested in eleven patients with advanced ATC with verified overexpression of PDGFR, of which eight had evaluable response [54]. The patients had a 6-month OS of 45% (95% CI: 16%–70%) and 6-month PFS of 36% (95% CI: 9%–65%). Twenty-five percent experienced PR and 50% SD. 

Lenvatinib, a multikinase inhibitor targeting VEGFR, FGFR, PDGFR, RET, and c-KIT was tested in a phase II, single-arm open-label study, which included 17 patients with ATC [55]. The study demonstrated an overall response rate of 24%, a median PFS of 7.4 months, and a median OS of 10.6 months. 

Sunitinib, a PDGFR and VEGFR inhibitor, was tested in a phase II trial in 71 patients with advanced radioactive iodine resistant TC, including a subgroup of four patients with advanced ATC [56]. Two of the patients did not undergo response evaluation for undisclosed reasons and were censored at 0 months. One patient experienced SD greater than 12 weeks. Median PFS was 9.8 months (95% CI: 7.8–11.9) for the two evaluated patients. 

### 2.3. BRAF V600E Inhibitors

Vemurafenib, a BRAF V600E inhibitor, was tested in a basket study of non-melanoma BRAF V600 mutated cancers [57]. The study included seven ATC patients, of which one experienced a CR (14%), one a PR (14%) and four experienced PD (57%), with an overall response of 29% (95% CI: 4–71). Median PFS and OS were not determined. One patient was missing response data. 

The combination of dabrafenib, a BRAF V600E inhibitor, and trametinib, a MEK1/2 inhibitor, was tested in a multi-center phase II trial which included 16 patients with advanced, BRAF V600E mutated ATC [58]. Interim data has been published showing an overall response rate of 69% (95% CI: 41%–89%). One patient experienced CR, 10 had PR, three had SD, one had PD. Seven responses were ongoing at the time of data cutoff. Of note, this is one of only two CRs reported to date in a targeted therapy trial in ATC. Median follow-up was 47 weeks (range 4–120). PFS and OS were not reached due to a lack of events, but 12-month estimates were 79% and 80%, respectively. This trial has paved the way for approval from the United States Food and Drug Administration (FDA) of this treatment combination for patients with BRAF V600E mutated ATC with locally advanced, unresectable, or metastatic ATC with no locoregional treatment options (approval granted May 2018). 

### 2.4. PI3K/mTOR Inhibitors

Everolimus, a mTOR inhibitor, has been tested in three phase II clinical trials which included subgroups of patients with ATC. The first two studies included a subgroup of six and an explorative cohort of seven ATC patients, neither producing an objective response [59,60]. The third included a subgroup of seven radioiodine refractory ATC patients, of which one experienced a near-CR and remained progression-free until 17.9 months. Two patients achieved SD and three PD [34]. Median PFS was 2.2 months (95% CI: 1–17), median OS was 4.6 months (95% CI: 1–29.9), and 2-year OS was 28.6% (95% CI: 4–61.2). Median follow-up time was 38.7 months, with one patient in the cohort still alive at last follow-up. In this study, 38 of 50 patients underwent targeted next-generation sequencing using OncoPanel_v2, and those with significant treatment responses were shown to have enrichment of the PI3K/mTOR/AKT pathway. Aside from the seven ATC patients, the study included 33 patients with differentiated TC and 10 with medullary TC. The median PFS of the 38 sequenced patients overall was 2.8 months, compared to 15.2 months for those with confirmed PI3K/mTOR/AKT mutations. The ATC patient with near-CR was found to have a nonsense mutation of the tumor suppressor gene *TSC2*, a negative regulator of the mTOR pathway. The patient subsequently acquired a novel *mTOR* mutation (*mTOR^F2018L^*) conferring resistance to everolimus. The patient with SD for 26 months was found to have an *NF1* and a *TP53* mutation. 

Studies with additional PI3K pathway inhibitors are currently lacking.

### 2.5. EGFR Inhibitors

Gefitinib, a selective EGFR inhibitor, was tested in a single-arm trial with 27 patients with advanced TC, five of which had ATC [61]. Results were poor, with four out of five patients progressing radiologically within two months of treatment. One patient treated with concurrent chemo-radiation experienced radiological SD (still present after 12 months) but developed symptoms from metastatic disease. 

### 2.6. Selective VEGR Inhibitors

Axitinib, a potent and selective pan-VEGFR inhibitor, was tested in 60 patients with advanced TC, of which two had ATC [62]. One of the two patients experienced a PR. 

### 2.7. Vascular-Targeting Agents

Fosbretabulin, a prodrug of the active molecule combretastatin A4 phosphate that targets tumor vasculature by destabilizing microtubules in endothelial cells, was tested in a multicenter, single-arm phase II trial in 26 patients with advanced ATC [63]. One patient had near partial remission after two treatment cycles, but rapidly progressed during the third cycle. The median OS was 4.7 months (95% CI: 2.5–6.4), with three patients however being alive at time of last follow-up at 12.1, 24.4 and 37.9 months. Additionally, a multicenter phase II randomized controlled trial, including 80 ATC patients with advanced disease, assigned patients 2:1 to the intervention arm (fosbretabulin, plus carboplatin and paclitaxel (F+CP)) or the control arm (carboplatin and paclitaxel (CP)) [64]. The results showed PR of 20% in the intervention group and 16% in the control group. No CR were observed. Median PFS was 3.3 months (95% CI: 2.3–5.6) for the intervention group and 3.1 months (95% CI: 2.7–5.4) for the control group. Median OS was greater, although not significantly, in the intervention arm (5.2 months, 95% CI: 3.1–9.0) compared to the control arm (4.0 months, 95% CI: 2.8–6.2). Median OS was, however, significantly better in two patient subgroups: a) those with larger tumors (>6 cm), with a median OS of 5.7 months (95% CI: 3.1–13.3) in the intervention arm, compared to 3.9 months (95% CI: 0.3–5.4) in the control arm, corresponding to a risk reduction of disease-related death of 27% (HR 0.73 (95% CI: 0.34–1.57)); and b) in younger patients (≤60 years) with a median OS of 10.6 months (95% CI: 3.1–13.3) in the intervention arm vs. 3.1 months (95% CI: 1.7–9.5) in the control arm, corresponding to a risk reduction of disease-related death of 62% (HR 0.38 (95% CI: 0.17–0.85)). Notably, this study is the only randomized controlled trial of targeted therapy in ATC patients to date.

### 2.8. Follow-Up and Treatment Discontinuation

Nearly half of trials did not report follow-up times, and a proportion were missing outcome data on patients alive at the end of the study (Supplementary Table S1 provides a detailed overview of reported follow-up, loss to follow-up, and treatment discontinuation). A significant number of patients in the included studies were lost to follow-up (ranging from 0–27.3%). Furthermore, treatment discontinuation across studies was high, with a significant number of patients discontinuing treatment due to disease progression. 

## 3. Adverse Events

The included clinical trials reported treatment-related adverse events (TrAE) and adverse events (AE) in general, using Common Terminology Criteria for Adverse Events v3.0-4.03 (CTCAE), and with grades ranging from I–V (mild, moderate, severe, life-threatening or death-inducing, respectively). Reporting of adverse events was highly variable across trials. Out of the 17 included studies, nine reported AEs in general, while eight reported TrAEs. Some studies reported the percentage of patients experiencing a specific grade of toxicity, while others reported how many patients experienced a specific TrAE/AE. Some studies reported the prevalence of each grade specifically, while others grouped grades (i.e., I–II, II–IV, etc.). Moreover, in trials including patients with different types of TC, the TrAEs/AEs were not stratified by TC subtype. Overall, TrAEs were common for all agents tested, but the majority of adverse events were mild (grade I–II toxicities), with relatively few grade III and, in particular, grade IV–V toxicities, as summarized in Table 3. Only two potentially treatment-related deaths were reported: one in a patient that died due to a malfunctioning tracheostomy tube potentially related to fosbretabulin treatment [64], and the other in a patient who died of a tumor-associated hemorrhage defined as possibly, probably or definitely related to pazopanib treatment [53]. 

Toxicity-related dose reductions were common and seen in up to 88 % of cases, but were not reported by every study. The greatest numbers of dose reductions were seen in patients treated with lenvatinib (88%), sorafenib (up to 78%) and everolimus (up to 62%). Treatment-related discontinuations were relatively few, although Ito Y et al. [51] reported discontinuation of sorafenib in 67% (although the majority of patients were able to restart treatment, and only 17% of patients had permanent discontinuation; the latter primarily due to transaminase increases) and Tahara et al. [55] reported discontinuation of lenvatinib in 65% of ATC patients. It is relevant to note that several of the studies included only a few ATC patients, and as such safety profiles could potentially differ if only ATC patients were considered. Nonetheless, the overall safety profile reported by the included studies was acceptable. 

## 4. Discussion

Clinical trials of targeted therapy in ATC have thus far primarily consisted of single-arm trials, and only six of the 18 included trials consisted of exclusively ATC patients, defined by relatively small groups of patients (range: *n* = 2–80, median *n* = 7). The low numbers of ATC patients enrolled in part reflects the rarity of ATC, and in part its rapid progression to death. These factors have made the design of larger randomized clinical trials challenging, underscoring the need for international multicenter initiatives. 

With regards to adverse events, reporting was not standardized across trials, making it difficult to compare safety profiles of drugs. It is important to note that although grade I and II toxicities are generally viewed as being acceptable, many ATC patients are enrolled at a late disease stage with low performance status where even milder toxicity may be problematic. Nevertheless, the overall safety profile of each targeted therapy agent tested was deemed acceptable. 

The overall outcomes of targeted therapy trials to date are generally disappointing with relatively few patients experiencing responses or SD. This may in part reflect a lack of adequate patient selection. Outcomes of targeted therapy trials may be improved by genetic profiling of patients prior to trial enrollment, as exemplified by the recent trial of combined dabrafenib–trametinib therapy in BRAF V600E mutated ATC [58]. This study reported one of the only two CRs to date in ATC, and yielded the best overall effect of any ATC targeted therapy trial with PR in 63% of patients and SD in 19%. These findings are supported by the results of a recent case series of 16 patients with advanced ATC [65]. Ten of these received lenvatinib, while six BRAF mutated patients received dabrafenib/trametinib treatment; the latter group showed improved survival compared to patients receiving lenvatinib. Median OS for all patients was 6.3 months (95% CI: 1.8–7.6); 3.9 and 9.3 months for the lenvatinib and dabrafenib-trametinib subgroups, respectively. Median PFS for all patients was 3.7 months (95% CI: 1.8–7.6); 2.7 and 5.2 months for the lenvatinib and dabrafenib-trametinib subgroups, respectively. The other CR reported in the included trials was also observed in the BRAF V600E mutated ATC setting from a phase II basket-study of vemurafenib [57]. There are additionally several case reports employing vemurafenib and achieving treatment responses in patients with BRAF V600E mutated ATC [66,67]. These results hold promise for the treatment of BRAF mutated ATC, and have paved the way for FDA approval of the dabrafenib-trametinib combination therapy in ATC. As a considerable proportion of ATC samples have been shown to co-harbor BRAF and PI3K pathway alterations, it will be further relevant to determine whether these PI3K pathway alterations confer intrinsic resistance to BRAF inhibition, as observed in BRAF-mutated melanoma.

The relevance of genetic profiling is further exemplified by the everolimus trial by Hanna et al., which showed significantly better outcomes in patients with PI3K/AKT/mTOR mutations than those without [34]. Given the relatively high frequency of activating PI3K/AKT/mTOR pathway alterations identified in ATC, there is strong rationale for testing additional inhibitors of this pathway. A current phase II trial is considering MLN0128, a mTOR inhibitor (NCT02244463). It may further be interesting to investigate the effect of lenvatinib, which has had documented PR as monotherapy, in combination with everolimus; a combination that is already FDA-approved for the treatment of advanced renal cell carcinoma [68]. 

Lenvatinib monotherapy produced a reported median OS of 10.6 months in one trial [55], suggesting a doubling of the current median OS for patients with ATC. However, Iyer et al., in a retrospective cohort that included ATC patients treated with lenvatinib, reported a modest OS of only 3.9 months [65]. Further trials are needed to clarify the impact of lenvatinib in this patient population, and whether certain patient subgroups benefit more significantly from this treatment. 

*ALK* alterations appear to be rare in ATC; however, they may be targetable. One case report of a 71-year old ATC patient with ALK overexpression secondary to an *ALK* gene rearrangement (both in the pulmonary metastases and the primary tumor) demonstrated a response of >90% across all pulmonary lesions during crizotinib treatment, which was sustained at 6 months of follow up [69]. A phase II trial is underway to test ceritinib in ATC patients with *ALK* alterations (NCT02289144). 

Similarly, EGFR inhibitors may be relevant in a small subset of patients with ATC. Masago et al. have described a case report of a patient with ATC with L858R mutated EGFR that was treated with erlotinib and a partial response was obtained, which was sustained at 6 months [70]. One patient in the gefitinib phase II trial also had long-term stable disease (>12 months) [61]. 

Furthermore, the identification of cell cycle gene alterations, such as CCNE1 amplifications, suggest that CDK inhibitors (such as CDK4/6 inhibitors) may be a viable treatment option. Preclinical studies in ATC cell lines and ATC xenograft models further support this [71,72,73]. These findings remain to be extended to clinical trials. 

There is a multitude of additional preclinical and early clinical studies that have shown promising results, including studies with aurora kinase inhibitors, epigenetic modifying strategies, compounds that can induce re-differentiation (such as retinoic acid), apoptosis enhancers, as well as a variety of combination therapy approaches. These strategies are beyond the scope of the current review; however, they are reviewed by Saini et al. [74] and Catalano et al. [75].

The clinical trials to date have enrolled patients that have experienced treatment failure after surgical resection and/or chemoradiation. It may be interesting for future studies to consider targeted therapy at an earlier stage and/or as part of multimodal treatment. A current phase II randomized controlled trial is exploring such a strategy evaluating pazopanib with paclitaxel in combination with intensity-modulated radiotherapy (NCT01236547). An additional trial is exploring sorafenib as neo-adjuvant treatment to potentially provide patients with an opportunity for surgery (NCT03565536) and a third trial is considering paclitaxel in combination with trametinib in patients with metastatic or local-regional disease not resectable for cure (NCT03085056).

Although not discussed in the current systematic review as no clinical trials have been published to date, immunotherapy may be relevant for a subgroup of ATC patients. PD-L1 has been shown to be highly expressed in ATC tumors [76,77], and ATC tumors are highly infiltrated by macrophages [78], resulting in an immunosuppressive tumor microenvironment. Furthermore, amplification of tumor evasive genes such as *PD-L1*, *PD-L2* and *JAK2* has been identified in a subset of ATC tumors (5%) [25], and mismatch repair deficiency has been reported in up to 36% of tumors [24,25,29,35]. Prior studies indicate that these alterations are associated with an improved response to immune checkpoint inhibitors such as nivolumab [79,80,81]. Encouraging data comes from a case report of a 62-year-old patient, with BRAF V600E mutated, PD-L1 positive ATC with lymph node and lung metastases, who was treated with vemurafenib and nivolumab. Vemurafenib had to be discontinued due to toxicity but nivolumab monotherapy was continued, and the patient experienced complete radiographic and clinical remission, still present 20 months after the start of nivolumab treatment [82]. Similarly, Cabanillas et al. reported on a 60-year old man with BRAF V600E mutated ATC treated with dabrafenib/trametinib on which he achieved partial response. The patient subsequently developed enlargement of a supraclavicular lymph node which showed >95% PD-L1 positivity, thus pembrolizumab was started. The patient underwent surgical resection and EBRT, and thereafter continued treatment with dabrafenib/trametinib/pembrolizumab, and was doing well at 11 months follow-up. Further promising data comes from a case series where salvage pembrolizumab was added to kinase inhibitor therapy at the time of progression (*n* = 12) resulting in OS ranging from 5.4 to 40.1 months (median OS 10.4 months, from the start of kinase inhibitor therapy) [83]. Kinase inhibitor therapy consisted of dabrafenib/trametinib or trametinib alone or lenvatinib alone. These data support testing of immunotherapy in clinical trials in ATC, and multiple trials are underway to address this. Pembrolizumab is being tested in metastatic and locally advanced anaplastic or undifferentiated TC (NCT02688608). Atezolizumab is being tested in combination with targeted therapies or taxanes in patients with ATC and PDTC (NCT03181100). Nivolumab combined with ipilimumab is being tested in an exploratory cohort of ATC patients (NCT03246958). 

## 5. Materials and Methods 

**Figure 2:**
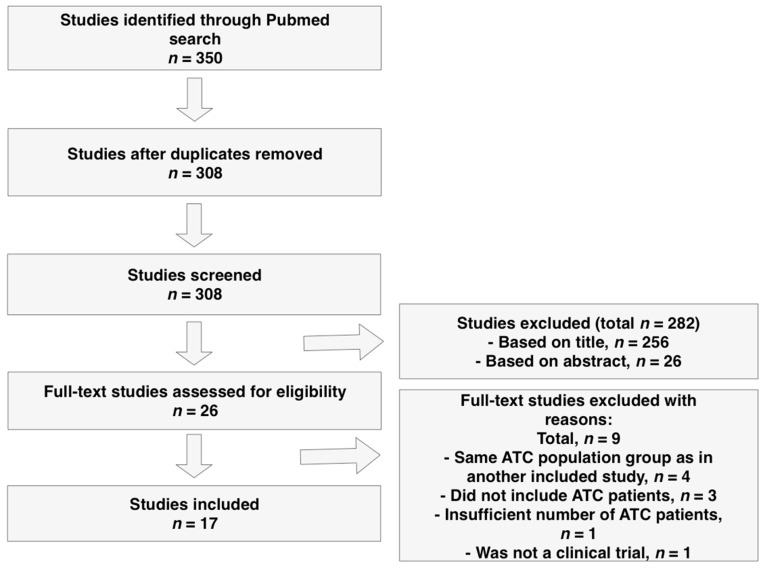
Flow diagram for study selection.

Relevant studies were identified through a systematic search in PubMed (Figure 2) using the search terms listed in Table 4. The following inclusion criteria were used: phase II or III clinical trials, human studies and cohorts with a minimum of two ATC patients. The date last searched was 25 May 2019. One author (JL) independently reviewed the publications. Studies were included based on the PRISMA 2009 guidelines. The number and type of patients included in each clinical trial, outcome data (treatment response, OS, PFS), length of follow-up, details regarding discontinuation of treatment, and data regarding adverse events were extracted from each study.

## 6. Conclusions

ATC is a rare cancer with a dire prognosis despite multimodal treatment. The characterization of the molecular landscape of ATC has suggested several potential therapeutic targets. The majority of targeted therapy trials to date have consisted of small single-arm studies and have presented modest results; however only few studies have selected or stratified patients by molecular alterations. In the setting of BRAF V600E mutated ATC, combined dabrafenib/trametinib treatment and vemurafenib have both demonstrated efficacy, and everolimus has provided promising results in patients with PI3K/mTOR/AKT pathway alterations. These studies underscore the importance of molecular profiling of tumors for appropriate patient selection and determination of genomic correlates of response. Additional rare targetable alterations have been identified, such as *ALK* and *EGFR* alterations, and warrant further exploration. Clinical trials testing targeted therapy as part of multimodal treatment, and in combination with immunotherapy, are underway. 

## Figures and Tables

**Table 1:** Overview of the frequency of the most common alterations in ATC, based on next generation sequencing studies.

Alteration	Latteyer [33]	Landa [24]	Pozdeyev [25]	Jeon [28]	Kunstman [29]	Hanna [34]	Tiedje [30]	Bonhomme * [31]	Khan [32]	Ravi [35]	Overall
	(*n* = 30)	(*n* = 33)	(*n* = 196)	(*n* = 11)	(*n* = 22)	(*n* = 7)	(*n* = 118)	(*n* = 144)	(*n* = 90)	(*n* = 14)	
***TERT* promoter mutation**	NA	73%	65%	NA	NA	NA	73%	54%	32%	36%	32–73%
**RAS-RAF-MAPK pathway**											
*BRAF*	7%	45%	41%	91%	27%	29%	11%	14%	34%	18%	7–91%
*RAS*	23%	24%	27%	9%	27%	14%	20%	43%	26%	18%	9–43%
**PI3K-AKT-mTOR pathway**											
*PIK3CA*	NA	18%	14%	18%	8%	0%	12%	6%	12%	18%	0–18%
*PTEN ^¶^*	NA	15%	11%	9%	0%	0%	0%	9%	13%	18%	0–18%
**SWI/SNF complex**	NA	36%	18%	NA	9%	14%	NA	1%	6%	27%	1–36%
**Histone methyl-transferases**	NA	24%	19%	18%	5%	0%	NA	NA	3%	54%	0–54%
**Mismatch repair**	NA	12%	4%	NA	27%	0%	NA	0%	2%	36%	0–36%
**Additional genes**											
*TP53*	60%	73%	65%	73%	27%	43%	55%	54%	66%	55%	27–73%
*ATM*	NA	9%	4%	0%	0%	57%	NA	4%	NA	27%	0–57%
*EIF1AX*	NA	9%	NA	0%	14%	0%	NA	N/A	NA	0%	0–14%
*NF1*	37%	9%	9%	0%	9%	14%	NA	NA	12%	9%	0–37%
*NF2*	NA	6%	12%	27%	5%	0%	NA	NA	14%	18%	0–27%
*RB1*	NA	9%	7%	0%	0%	0%	0.8%	1%	6%	18%	0–18%
*TSHR*	NA	6%	3%	0%	5%	0%	NA	NA	NA	9%	0–9%
*STK11*	NA	6%	0%	0%	0%	0%	NA	1%	NA	9%	0–9%
ALK ^†^	20%	0%	0%	0%	0%	0%	0%	1%	2%	0%	0–20%
*CCNE1 ^#^*	NA	NA	4%	NA	NA	NA	NA	NA	4%	29%	4–29%

TERT = telomerase reverse transcriptase, NA = Not available. * Next-generation sequencing results interpretable for 94 (65.3%) cases, and Sanger sequencing (*TERT*) for 98 (68.1%) cases. *^¶^* Primarily *PTEN* loss-of-function mutations are reported; however, fusions are also reported. *^†^* Both *ALK* fusions and mutations reported. ^#^ Amplifications of *CCNE1* reported.

**Table 2:** Efficacy results in phase II clinical trials of targeted therapy in ATC.

Trial	Tested Agent	Design	No. of ATC Patients	PR	CR	SD	PD	PFS	OS
**Kloos** [49]	Sorafenib	Single arm	4 (out of 56)	0%	0%	25%	75%	NA	NA
**Savvides** [50]	Sorafenib	Single arm	20	10%	0%	25%	NA	1.9 months (95% CI: 1.3–3.6 months)	3.9 months (95% CI: 2.2–7.1 months)
**Ito** [51]	Sorafenib	Single arm	10 (out of 18)	NA	NA	40%	60%	2.8 months (95% CI: 0.7–5.6)	5 months (95% CI: 0.7–5.7)
**Sherman** [52]	Sorafenib/ Temsirolimus	Single arm	2 (out of 36)	50%	0%	0%	50%	NA	NA
**Bible** [53]	Pazopanib	Single arm	15	0%	0%	0%	100%	2 months	3.7 months
**Ha** [54]	Imatinib	Single arm	8 (11 total, but 8 evaluable)	25%	0%	50%	NA	6 months: 36% (95% CI: 9–65) 12 months: 24.2% (95% CI: 3.8–54.1) 18 months: 12.1% (95% CI: 0.7–41.1)	6 months: 45% (95% CI: 16–70)
**Tahara** [55]	Lenvatinib	Single arm	17 (out of 51)	24%		71%	6%	7.4 months (95% CI: 1.7–12.9)	10.6 months (95% CI: 3.8–19.8)
**Ravaud** [56]	Sunitinib	Single arm	4 (out of 71)	0%	0%	25%	NA	9.8 months (95% CI: 7.8–11.9)	NA
**Hyman** [57]	Vemurafenib	Single arm	7 (out of 122)	14%	14%	0%	57%	NA	NA
**Subbiah** [58]	Dabrafenib + Trametinib	Single arm	16 (out of 100)	63%	6%	19%	6%	79% *	80% *
**Lim** [59]	Everolimus	Single arm	6 (out of 38)	0%	0%	NA	NA	2.5 months (95% CI: 4.8–16.0)	NA
**Schneider** [60]	Everolimus	Single arm	7 (out of 35)	0%	0%	0%	100%	3.7 months	4.7 months
**Hanna** [34]	Everolimus	Single arm	7 (out of 50)	14%	0%	29%	43%	2.2 months (95% CI: 1–17.9)	4.6 months (95% CI: 1–29.9)
**Pennell** [61]	Gefitinib	Single arm	5 (out of 27)	0%	0%	20%	NA	NA	NA
**Cohen** [62]	Axitinib	Single arm	2 (out of 60)	50%	0%	0%	50%	NA	NA
**Mooney** [63]	Fosbretabulin	Single arm	26	0%	0%	27%	58%	NA	Median OS: 4.7 months 6 months: 34% 12 months: 23%
**Sosa** [64]	Fosbretabulin + Carboplatin/ Paclitaxel (FCP) vs. Carboplatin/ Paclitaxel (CP)	RCT	80	FCP = 20% CP = 16%	0%	FCP = 40% CP = 44%	FCP = 49% CP = 52%	FCP = 3.3 months (95% CI: 2.3–5.6) CP = 3.1 months (95% CI: 2.7–5.4)	Median OS: FCP = 5.2 months (95% CI: 3.1–9.0) CP = 4.0 months (95% CI: 2.8–6.2) 1-year OS: FCP = 26% CP = 9%

PR = partial response, CR = complete response, SD = stable disease, PD = progressive disease, PFS = progression-free survival, OS = overall survival. * PFS and OS not reached due to lack of events. The numbers represent 12 months estimates.

**Table 3:** Overview of adverse events (AEs) and treatment-related adverse events (TrAE) in phase II trials testing targeted therapy in ATC patients.

Trial	No. of Patients	Grade of TrAE/AE					Any Grade	DR	TD
		I	II	III	IV	V			
**Kloos** [49]	56 (4 ATC)	VM		NA	NA (but few)	2%, likely unrelated to treatment	NA	52%	NA
**Savvides** [50]	20	VM		NA	5%	0%	NA	NA	NA
**Ito** [51]∆	18 (10 ATC)	NA	NA	NA	NA	17%, unrelated to treatment	NA	78%	67% (only 17% permanent)
**Sherman** [52]	36 (2 ATC)	NA	NA	64% grade 3 or greater		3%	NA	61%	14% (an additional 17% discontinued only sorafenib)
**Bible** [53]	15	>70%		>10%		6.7%, possibly related	NA	27%	11%
**Ha** [54]∆	11	>70%		>25%	0%	0%	NA	27%	0%
**Tahara** [55]∆	51 (17 ATC)	VM		77%	6%	8%, unrelated to treatment	100%	88%	65%
**Ravaud** [56]∆	71 (4 ATC)	NA		>25%	>1%	>7%	>80%	NA	NA
**Hyman** [57]∆	122 (7 ATC)	NA		43% (for ATC cohort)		NA	86% (for ATC cohort)	NA	NA
**Subbiah** [58]∆	100 (16 ATC)	NA		50% (for ATC cohort)		NA	94% (for ATC cohort)	30% (entire cohort, *n* = 100)	8% (entire cohort, *n* = 100)
**Lim** [59]∆	38 (6 ATC)	>84%		>15%	2%	2%, unrelated to treatment	NA	NA	2%
**Schneider** [60]∆	35 (7 ATC)	Safety data not provided for ATC patients.							
**Hanna** [34]	50 (7 ATC)	50%		42%	2%	0%	94%	62%	NA
**Pennell** [61]∆	27 (5 ATC)	NA	NA	11%	0%	0%	NA	NA	7 %
**Cohen** [62]	60 (2 ATC)	NA	NA	35%		NA	93%	42%	7%
**Mooney** [63]∆	26	VM	NA	35%	4%	0%	NA	NA	4 %
**Sosa** [64]	80	FCP = 27.5% CP = 29.2%		FCP = 25.5% CP = 29.2%	FCP = 37.3% CP = 16.7%	1%, possibly related to treatment	NA	NA	1%

DR = dose reduction, TD = treatment discontinuation due to TrAEs, NA = not applicable or not reported, VM = precise number not declared but vast majority, FCP = fosbretabulin + carboplatin/paclitaxel, CP = carboplatin/paclitaxel, ∆ These trials have not reported TrAEs but rather reported AEs in general.

**Table 4:** Search strings used in systematic search on PubMed.

Search String	Filters Used	Search Results
1. MESH term: “Thyroid Neoplasms/drug therapy”	Clinical trials	269
2. “Anaplastic thyroid cancer”	Clinical trials	20
3. “Anaplastic thyroid carcinoma”	Clinical trials	21
4. “Anaplastic thyroid cancer” OR ”Anaplastic thyroid carcinoma”) AND (”Phase 2” OR ”Phase II” OR ”Phase 3” OR ”Phase III”)	None	40

## Supplementary Materials

The following are available online at https://www.mdpi.com/2072-6694/11/7/943/s1, Table S1: Reported follow-up, loss to follow-up, and treatment discontinuation in the included trials.
