# Supplementary material for: A Systematic Review of Phase II Targeted Therapy Clinical Trials in Anaplastic Thyroid Cancer
Source: Cancers (Basel). 2019 Jul 4;11(7):943. doi: 10.3390/cancers11070943 (PMC6678800; doi:10.3390/cancers11070943)
Supplement: Supplementary file 1 [file cancers-11-00943-s001.pdf]

Supplementary Materials: Systematic Review of Advances in Targeted Therapy for Anaplastic Thyroid Cancer

Table S1. Reported follow-up, loss to follow-up, and treatment discontinuation in the included trials.

	Follow Up Reported	No. of Patients	Unavailable for Response Evaluation	No. of Treatment Discontinuations
**Kloos [49]**	No	56 (4 with ATC)	**2 patients (3.4 %)**<br>- Unreported reasons	**54 patients (93 %)**<br>- 35 due to progressive disease<br>- 14 due to AEs<br>- 1 due to other unreported reasons
**Savvides [50]**	No	20	**2 patients (10 %)**<br>- Due to disease progression	NA
**Ito [51]**	No	18 (10 with ATC)	None	**3 patients (17 %)**<br>- Due to AEs
**Sherman [52]**	No	36 (2 with ATC)	**6 patients (17 %)**	**35 patients (97 %)**<br>**-** 12 due to withdrawal of consent<br>- 10 due to progressive disease<br>- 5 due to excessive toxicity
**Bible [53]**	No	16	**1 patient (6.25 %)**<br>- Cancellation before treatment for unknown reasons	**15 patients (100 %)**<br>- 12 due to progressive disease<br>- 1 due to death<br>- 2 due to AEs
**Ha [54]**	Yes	11	**3 patients (27.3 %)**<br>- Lack of radiological evaluation	**1 patient (9 %):**<br>- AE not related to the treatment or disease
**Tahara [55]**	No	60 (17 with ATC)	**9 patients (15 %)**<br>- Did not meet inclusion criteria	**13 out of 17 ATC patients (77 %)**<br>- 12 due to progressive disease<br>- 1 due to suicide attempt
**Ravaud [56]**	Yes	71 (4 with ATC)	**6 patients (8 %)**<br>(two with ATC)	**62 patients (87 %)**<br>- 38 due to death<br>- 18 due to loss to follow-up
**Hyman [57]**	No*	122 (7 with ATC)	**2 patients (2 %)**<br>- Withdrew early, reason not reported	**At least 4 due to AE** (3 %)**
**Subbiah [58]**	Yes	16	None	**3 patients (19 %)**

				- 2 due to AE<br>- 1 due to PD
**Lim [59]**	Yes	40 (6 with ATC)	**5 patients (12.5 %)**<br>- 2 withdrew consent before treatment for unknown reasons<br>- Outcomes of 3 patients are missing for unknown reasons	**23 patients (58 %)**<br>- 2 withdrew consent before and 3 during treatment for unknown reasons<br>- 1 due to AE<br>- 14 due to disease progression
**Schneider [60]**	Yes	35	None	**1 patient (3 %)**<br>- Due to AE
**Hanna** [34]	Yes	50 (7 with ATC)	**6 patients (12 %)**<br>(1 with ATC)	**46 patients (92 %)**<br>- Progressive disease
**Pennel [61]**	Yes	27 (5 with ATC)	**2 patients (7.4 %)**<br>**-** Both due to AE	**2 patients (7.4 %)**<br>- The same two unavailable for evaluation
**Cohen [62]**	Yes	60 (2 with ATC)	**15 patients (25 %)**<br>- 8 did not meet response criteria<br>- 7 missing post baseline data for unknown reasons	**60 patients (100 %)**<br>For both studies:<br>- 20 had insufficient clinical response<br>- 14 planned to enroll in the ongoing extension study<br>- 11 due to non-fatal AEs<br>- 4 due to fatal AEs<br>- 9 refused further participation<br>- 2 lost to follow-up
**Mooney [63]**	Yes	26	None	**1 patient (4 %)**
**Sosa [64]**	No	80	**5 patients (6.25 %)**<br>- 2 due to patient refusal<br>- 1 did not meet inclusion criteria<br>- 1 due to investigators' decision	**50 patients (67 %)**<br>- 40 due to disease progression<br>- 6 withdrew or refused<br>- 2 due to AE<br>- 1 unreported reason

* Study still ongoing. ** Only reported for the cohort with Erdheim–Chester disease or Langerhans'cell histiocytosis.
